# Retrospective database analysis for clinical diagnoses commonly associated with pneumococcal diseases in the Malaysian healthcare system over a 3-year period (2013–2015)

**DOI:** 10.1186/s12879-023-08611-3

**Published:** 2024-01-12

**Authors:** Saravanan S. R. Sundaramurthy, Kristen E. Allen, Mark A. Fletcher, Kok Fui Liew, Boekhtiar Borhanuddin, Mohammad Ali, Graciela Morales, Bradford Gessner, Jerusha Naidoo, Jo Southern

**Affiliations:** 1 Malaysian Association for Family and Child Health (MAFCH), Kuala Lumpur, Malaysia; 2 Vaccines Medical and Scientific Affairs, Pfizer Biopharma, Collegeville, Pennsylvania USA; 3 Emerging Markets Region Medical Affairs, Pfizer Biopharma, New York City, USA; 4 Pfizer Biopharma, Kuala Lumpur, Malaysia

**Keywords:** Pneumococcal, Pneumonia, Meningitis, Septicaemia, Bacteraemia, Malaysia

## Abstract

**Background:**

Pneumococcal disease caused by *Streptococcus pneumoniae* is an important cause of morbidity and mortality across all ages, particularly in younger children and older adults. Here, we describe pneumococcal disease hospitalizations at Ministry of Health (MoH) facilities in Malaysia between 2013 and 2015.

**Methods:**

This was a retrospective databases analysis. Tabular data from the Malaysian Health Data Warehouse (MyHDW) were used to identify microbiologically confirmed, pneumococcal disease hospitalizations and deaths during hospitalization, using hospital-assigned ICD-10 codes (i.e., classified as meningitis, pneumonia, or non-meningitis non-pneumonia). Case counts, mortality counts, and case fatality rates were reported by patient age group and by Malaysian geographic region.

**Results:**

A total of 683 pneumococcal disease hospitalizations were identified from the analysis: 53 pneumococcal meningitis hospitalizations (5 deaths and 48 discharges), 413 pneumococcal pneumonia hospitalizations (24 deaths and 389 discharges), and 205 non-meningitis non-pneumonia pneumococcal disease hospitalizations (58 deaths and 147 discharges). Most hospitalizations occurred in children aged < 2 years. Crude mortality was highest among children aged < 2 years (for all three disease categories), among adults aged ≥ 65 years (for pneumococcal pneumonia), or among adults aged 65–85 years (for non-meningitis non-pneumonia pneumococcal disease). The case fatality rate, all ages included, was 5.8% for pneumococcal pneumonia, 9.1% for pneumococcal meningitis, and 28.3% for non-meningitis non-pneumonia pneumococcal disease.

**Conclusions:**

Our study is the first to document pneumococcal disease hospitalizations and deaths during hospitalization in Malaysia. Although this database analysis likely underestimated case counts, and the true disease burden could be even greater, the study demonstrates a substantial burden of pneumococcal disease. Public health measures, including vaccination, would significantly contribute to the prevention of hospitalizations and deaths associated with pneumococcal disease in Malaysia.

**Supplementary Information:**

The online version contains supplementary material available at 10.1186/s12879-023-08611-3.

## Background

*Streptococcus pneumoniae* is a common cause of serious infections, invasive or non-invasive, across the span of age. Invasive pneumococcal disease (IPD) includes meningitis, pneumonia (bacteremic pneumonia and empyema), and non-meningitis/non-pneumonia disease (bacteremia/septicemia). Pneumococcal meningitis, associated with 10–30% case-fatality, leaves up to 50% of survivors suffering neurological sequelae [1]. Pneumococcal infections can also cause mucosal (non-invasive) diseases, such as nonbacteraemic pneumonia or acute otitis media (AOM). Pneumococcal pneumonia, which has both invasive (bacteremic pneumonia and empyema) and non-invasive (nonbacteraemic pneumonia) presentations, is a major cause of morbidity and mortality worldwide [2–5].

Health care services in Malaysia consist of public sector, Ministry of Health (MoH)-run, primary health care centers and hospitals, and there are private health care services mainly located in physician clinics and in hospitals in urban areas. MoH-run, public sector, health care services are administered through central, state and district offices. In addition to regulating its own health care services, the MoH also regulates the private health care services, as well as the pharmaceutical industry and food safety. The Malaysian healthcare services maintain records across the population using individuals’ unique National Registration Identification Card (NRIC) numbers, allowing linkage of each individual's hospitalisation(s), laboratory result(s), and death certification.

Analyses of national health databases have been widely used to substantiate *S. pneumoniae* disease burden, notably in Taiwan, France, and the USA [6–8]. Although similar analyses generally have not been done in Southeast Asia due to the absence or lack of access to databases [9], the Malaysian health service NRIC numbers allow linkage of individual hospitalisation, laboratory, and death certification records.

The pediatric national immunisation programme (NIP) in Malaysia began in December 2020, after the surveillance period for this study [10]. The objective of this retrospective database analysis was to describe – before the introduction of pneumococcal vaccination for children below 2 years of age into the NIP – microbiologically confirmed pneumococcal diseases treated at MoH facilities in Malaysia for all ages from 2013-2015. It is the first study to document pneumococcal-associated hospitalizations and deaths at MoH hospitals in Malaysia.

## Methods

### Health data

MoH tabular data from the Malaysian Health Data Warehouse (MyHDW) from 2013–2015 (Health Informatics Centre, Planning Division, Ministry of Health Malaysia 2017) were provided in response to a request from this group. The dataset included ICD-10 codes, age of the patient (in discrete year-of-age format), Malaysian state where hospitalization occurred, mortality, discharge disposition (alive or deceased), and year of discharge. (Mortality describes death during hospitalization.) Based on further information by MyHDW, MI-Harmony was used “to harmonise data by codifying actual data with SNOMED CT codes and to perform semantic-based query over the SNOMED CT codified data”. MI-Harmony is basically an automated tool that harmonises the medical terms from diverse unstructured textual data from different sources into standardized medical terms, which will be used for the purpose of extraction of the required data from the database when needed. Due to the limitation of the system, there was a possibility that the case counts in the provided tabular data had included repeat episodes from the same individual, and the database did not specify whether the diagnosis associated with hospitalization was the primary or secondary reason for the hospitalization.

These MoH medical facility data of microbiologically confirmed pneumococcal diseases (i.e., meningitis, pneumonia, and non-meningitis/ non-pneumonia) were used to assess the case counts and crude disease incidence, as well as the mortality counts, case-fatality, and crude mortality incidence. The tabular data were disaggregated into individual-level datasets for the purpose of analysis in the current study. ICD-10 codes were classified as follows: pneumococcal meningitis (G00.1, Pneumococcal meningitis); pneumococcal pneumonia (J13, Pneumonia due to *Streptococcus pneumoniae*); or non-meningitis non-pneumonia pneumococcal disease (B95, *Streptococcus pneumoniae* as cause of diseases classified elsewhere; A40.3, Sepsis due to *Streptococcus pneumoniae*). This dataset was from a passive national data collection system for which microbiologically confirmed pneumococcal disease case counts were available, although no data were reported from the system on pneumococcal serotypes or on the frequency of laboratory specimens being tested for serotype. (Published serotyping results from pneumococcal isolates in Malaysia are available from governmental and academic sources; please see Discussion for details.)

### Population data

**Fig. 1:**
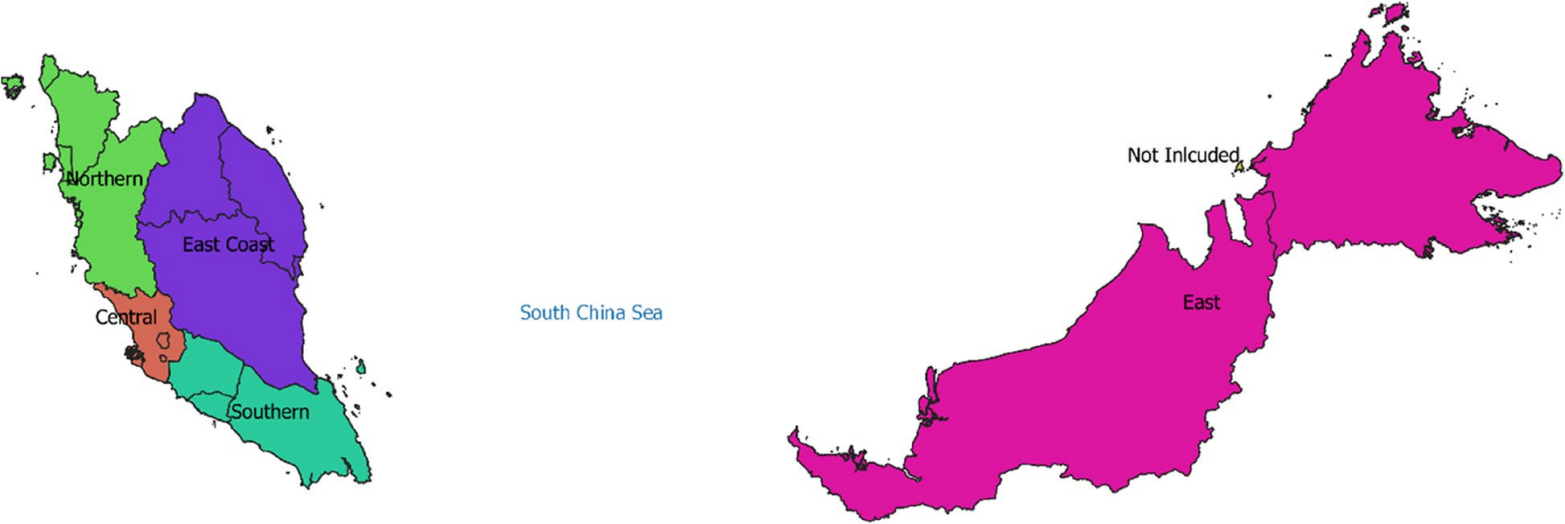
Regions of Malaysia as defined in the study (W.P. Labuan State is not included in this study). Accessed at—Humanitarian Data Exchange (https://data.humdata.org/dataset/malaysia-administrative-level-0-2-boundaries), and are shared under CC by license

Population data for each of the three study years, 2013–2015, by state and by age group, were obtained from the Department of Statistics Malaysia Official Portal (dosm.gov.my). States were categorized within five geographic regions as: i) Northern (Perlis, Kedah, Pulau Pinang, and Perak); ii) Central (Selangor, W.P. Kuala Lumpur and W.P. Putrajaya); iii) Southern (Negeri Sembilan, Melaka, and Johor); iv) East Coast (Kelantan, Terengganu, and Pahang); and v) East (Sabah and Sarawak) (Fig. 1). (No case data were available for the state of Perlis in the Northern region.) The age groups in discrete, year-of-age format were < 2 years, 2 to < 5 years, 5 to < 18 years, 18 to < 65 years, 65 to < 85 years, and 85 + years.

### Statistical methods

The final analysis used the population and case (individual-level) datasets for all three years, 2013–2015. (One case was removed from the dataset due to missing age at admission.) Data cleaning and statistical analyses were carried out using SAS Studio (SAS Institute Inc., Cary, NC, USA).

For these microbiologically confirmed cases treated at MoH facilities, the case counts, crude incidence (per 100,000 persons), fatality counts, case-fatality rates, and crude mortality rates (per 100,000 persons) were calculated and presented by age group and by geographic region of the country. Crude incidence rates and mortality rates were calculated using number of cases or deaths from MoH-run primary health care centres and hospitals as the numerator, and the mid-year population, overall and for each age group or region of the country, as the denominator. Crude incidence was also calculated for each of the three pneumococcal disease categories (pneumococcal meningitis, pneumococcal pneumonia, and non-meningitis non-pneumonia pneumococcal disease). Case fatality rates were calculated as the number of deaths divided by the total number of cases for each category, multiplied by 100. The exact Poisson method was used to compute the 95% confidence intervals for the rates. 

## Results

Over the three study years, using the selected ICD-10 codes denoting microbiologically confirmed pneumococcal disease that had been treated in Malaysian MoH facilities, there were 53 pneumococcal meningitis hospitalizations (5 deaths and 48 discharges), 413 pneumococcal pneumonia hospitalizations (24 deaths and 389 discharges), and 205 non-meningitis non-pneumonia pneumococcal disease hospitalizations (58 deaths and 147 discharges).

### Case counts by age group

**Fig. 2:**
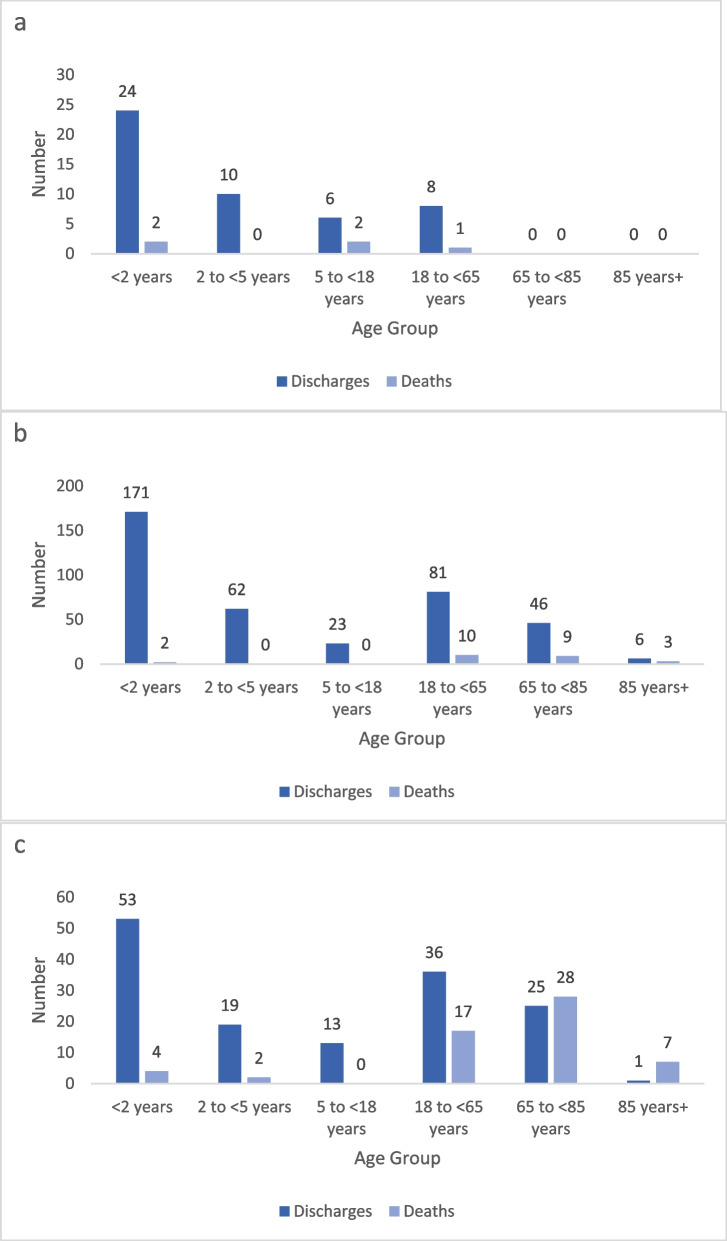
**a** Pneumococcal meningitis hospital discharges and deaths by age group, Malaysia 2013–2015. **b** Pneumococcal pneumonia hospital discharges and deaths by age group, Malaysia 2013–2015. **c** Non-meningitis non-pneumonia pneumococcal disease hospital discharges and deaths by age group, Malaysia 2013–2015

Most hospitalizations occurred in the < 2 years age group, whether for pneumococcal meningitis (49.1%, 26/53), pneumococcal pneumonia (41.9%, 173/413), or non-meningitis non-pneumonia pneumococcal disease (27.8%, 57/205) (Fig. 2a-c).

### Crude incidence

The crude incidence for pneumococcal meningitis in children < 2 years of age was 1.03 per 100,000 [95% CI 0.71, 1.46]. (Additional crude incidence rates by age group are available in the Supplementary Table S1).

### Fatality counts by age group

The non-meningitis non-pneumonia pneumococcal disease category had 58 deaths, which was the largest number of deaths across the three disease categories. Among these 58 deaths, most occurred in adults (77.6%, 45/58), in the 65- to < 85-year-old age group (48.3%, 28/58) or in the 18- to 65-year-old age group (29.3%, 17/58) (Fig. 2c).

### Case fatality rates

In our study, the overall case fatality rates were 9.1% (5/53) for pneumococcal meningitis, 5.8% (24/413) for pneumococcal pneumonia, and 28.3% (58/205) for non-meningitis non-pneumonia pneumococcal disease. Across all geographic regions, the highest case fatality rate was seen for pneumococcal meningitis in the 5 to < 18-year-old age group (25.0%, 2/8), while the 85 + year age group had the highest case fatality rates of pneumococcal pneumonia (33.3%, 3/9) and of non-meningitis non-pneumonia pneumococcal disease (87.5%, 7/8) (Table 1).

**Table 1:** Case fatality rate, death/cases (%), of pneumococcal meningitis^1^, pneumococcal pneumonia^2^, and non-meningitis non-pneumonia pneumococcal disease^3^ by geographic region and age group, Malaysia 2013–2015

Age, years							
**Region**	** < 2 years**	**2 to < 5 years**	**5 to < 18 years**	**18 to < 65 years**	**65 to < 85 years**	**85 years + **	**All ages**
**Pneumococcal meningitis**							
Northern^a^	1/2 (50.0)	0/2 (0)	0/2 (50.0)	0/1 (0)	0/0 (0)	0/0 (0)	1/7 (14.3)
Central^b^	1/8 (12.5)	0/0 (0)	0/1 (0)	0/1 (0)	0/0 (0)	0/0 (0)	1/10 (10.0)
Southern^c^	0/6 (0)	0/3 (0)	1/2 (50.0)	0/1 (0)	0/0 (0)	0/0 (0)	2/12 (16.7)
East Coast^d^	0/1 (0)	0/0 (0)	0/0 (0)	0/0 (0)	0/0 (0)	0/0 (0)	0/1 (0)
East^e^	0/9 (0)	0/5 (0)	1/3 (33.3)	0/6 (0)	0/0 (0)	0/0 (0)	1/23 (4.4)
All regions	2/26 (7.7)	0/10 (0)	2/8 (25.0)	1/9 (11.1)	0/0 (0)	0/0 (0)	5/53 (9.1)
**Pneumococcal pneumonia**							
Northern^a^	0/15 (0)	0/3 (0)	0/0 (0)	0/4 (0)	1/3 (33.3)	0/1 (0)	1/26 (3.9)
Central^b^	0/91 (0)	0/33 (0)	0/8 (0)	6/51 (11.8)	7/35 (20.0)	3/6 (50.0)	16/224 (7.1)
Southern^c^	1/24 (4.2)	0/10 (0)	0/5 (0)	1/7 (14.3)	1/2 (50.0)	0/1 (0)	3/49 (6.1)
East Coast^d^	0/14 (0)	0/8 (0)	0/4 (0)	1/17 (5.9)	0/5 (0)	0/0 (0)	1/48 (2.1)
East^e^	1/29 (3.5)	0/8 (0)	0/6 (0)	2/12 (16.7)	0/10 (0)	0/1 (0)	3/66 (4.6)
All Regions	2/173 (1.2)	0/62 (0)	0/23 (0)	10/91 (11.0)	9/55 (16.4)	3/9 (33.3)	24/413 (5.8)
**Non-meningitis non-pneumonia pneumococcal disease**							
Northern^a^	1/3 (33.3)	0/1 (0)	0/2 (0)	1/4 (25.0)	1/4 (25.0)	0/0 (0)	3/14 (21.4)
Central^b^	1/16 (6.3)	0/6 (0)	0/2 (0)	8/29 (27.6)	15/31 (48.4)	0/7 (0)	31/91 (34.1)
Southern^c^	0/3 (0)	1/2 (50.0)	0/2 (0)	4/9 (44.4)	4/7 (57.1)	0/1 (0)	9/24 (37.5)
East Coast^d^	2/4 (50.0)	0/1 (0)	0/1 (0)	1/4 (25.0)	1/2 (50.0)	0/0 (0)	4/12 (33.3)
East^e^	0/31 (0)	1/11 (9.1)	0/6 (0)	3/7 (42.9)	7/9 (77.8)	0/0 (0)	11/64 (17.2)
All Regions	4/57 (7.0)	2/21 (9.5)	0/13 (0)	17/53 (32.1)	28/53 (52.8)	7/8 (87.5)	58/205 (28.3)

95% CIs for proportions were calculated using exact method (Clopper-Pearson)

^1^ICD-10 code: G00.1 (Pneumococcal meningitis)

^2^ICD-10 code: J13 (Pneumonia due to Streptococcus pneumoniae)

^3^ICD-10 code: B95.3 (Streptococcus pneumoniae as cause of diseases classified elsewhere), A40.3 (Sepsis due to Streptococcus pneumoniae)

^a^Kedah, Pulau Pinang, Perak

^b^Selangor, W.P. Kuala Lumpur, W.P. Putrajaya

^c^Negeri Sembilan, Melaka, Johor

^d^Kelantan, Terengganu, Pahang

^e^Sabah, Sarawak

### Crude mortality incidence

Crude mortality rates were high among children aged < 2 years for all three disease categories, among adults > 65 years for pneumococcal pneumonia, and among adults aged 65 to < 85 years for non-meningitis non-pneumonia pneumococcal disease. (Additional crude mortality rates are in the Supplementary Table S2).

## Discussion

The case counts and the case-fatality rates in this analysis varied across age groups in accordance with expectations from global disease surveillance, with greater values observed in the youngest and oldest age groups. Variation in case fatality rates associated with various manifestations of IPD in Malaysia was also evident by geographic region (Table 1). Pneumococcal meningitis case fatality rates ranged from 0% in the East Coast to 16.7% in the Southern region; for pneumococcal pneumonia the range was from 2.1% in the East Coast region to 7.1% in the Central region; and for non-meningitis non-pneumococcal disease it was from 17.2% in the East region to 37.5% in the Southern region. In addition to age group and geographic region, other sources of variation, which were not measured, could have been individual risk factors for pneumococcal infection, age-specific exposure to *Streptococcus pneumoniae* transmission, access to healthcare services or medical technologies, or social factors (such as rural versus urban lifestyles) (Department of Statistics Malaysia Official Portal [dosm.gov.my]).

Calculated incidence rates and mortality rates substantially underestimated the burden of disease. In the numerator, the number of cases was based on microbiological confirmation of a pneumococcal infection, although it cannot be assumed that all pneumococcal infections were microbiologically confirmed. State populations were used as the denominator, but this may not have reflected an accurate estimation of the catchment areas for MoH-run primary health care centres and hospitals. For instance, the East geographic region (Sabah and Sarawak) is predominately an undeveloped rural area, marked by health literacy issues and challenges in access to healthcare, where some villages are only accessible by water route. Furthermore, Malaysia is served by both public health and private sector systems, and the relative patient proportion for each by state was not possible to ascertain.

In contrast to our study, where the crude incidence for pneumococcal meningitis in children < 2 years of age was estimated to be 1.03 per 100,000 [95% CI 0.71, 1.46], in studies from the pre-PCV era that consistently obtained cultures from suspected cases within well-defined catchment areas, the reported incidence ranged from 10 per 100,000 in industrialized countries to greater than 100 per 100,000 in developing country settings [1, 11]. Further suggesting the likely underestimation of disease in the current study, a prospective cohort study in children aged < 5 years who lived in Nha Trang, Vietnam measured the incidence of IPD as at least 48.7 cases per 100,000 children (95% CI, 27.9–85.1) [12].

Other studies in the region include a 2015 study of pneumococcal pneumonia in Southeast Asia that estimated the incidence of pneumococcal pneumonia at 2,432 per 100,000 children aged < 5 years, causing an estimated 69,200 deaths [13]. In Thailand, a study in 2007 documented that the highest incidences of IPD occurred in children aged < 5 years (8.8–12.3 per 100,000 individuals) and in adults aged ≥ 75 years (26 per 100,000 individuals) [14]. Another study in Thailand of two rural provinces from 2005–2010 documented the annual incidence of hospitalization due to pneumococcal bacteremia at 3.5 per 100,000 person-years. The highest rates of hospitalization occurred in infants aged < 1 year (33.8 per 100,000 person-years), children aged < 5 years (11.1 per 100,000 person-years), and adults aged ≥ 65 years (13.6 per 100,000 person-years) [15]. Of note was the authors’ caveat that these estimated incidence rates may be substantial underestimates of the true burden of pneumococcal bacteremia in Thailand because most patients were likely diagnosed in an outpatient setting without hospitalization [14]. Another Thailand study conducted in the same provinces from 2006 to 2011 found that the annual incidence of hospitalization due to pneumococcal pneumonia in adults was 30.5 cases per 100,000 person-years, with the highest rates of hospitalization in adults aged ≥ 70 years (150 per 100,000 person-years) [16].

For the crude mortality rates, it is also likely that the numerator was undercounted because most fatalities associated with pneumococcal infections probably had not been confirmed microbiologically, while the state population denominator would have overestimated the catchment area for the MoH-run primary health care centres and hospitals. Within our study, the crude mortality rates in the < 2 years age group was 0.06 per 100,000 [95% CI 0.01, 0.23] for pneumococcal meningitis and 0.06 per 100,000 [95% CI 0.01, 0.23] for pneumococcal pneumonia, and it was 0.13 per 100,000 [0.04, 0.33] for non-meningitis non-pneumonia pneumococcal disease. By contrast, higher population-based mortality rates were estimated for the Western Pacific region in young children (1–59 months) in the year 2000 (i.e., pre-PCV era): 3 per 100,000 for pneumococcal meningitis, 28 per 100,000 for pneumococcal pneumonia, and 3 per 100,000 for non-pneumonia non-meningitis pneumococcal disease [3].

Notably in North America and Europe, national health databases have been widely analyzed to substantiate *S. pneumoniae* disease burden [6–8], although similar analyses generally have not been done in Southeast Asia, due to the absence or lack of access to databases [9]. This study adds to the available pneumococcal disease burden evidence in Malaysia. A review study of IPD in Asia [17] identified only two relevant studies conducted in Malaysia, both now many years ago. The first paper assessed circulating, disease-causing pneumococcal serotypes in relation to those included in PPSV23, using data from 1994–1995, and concluded that vaccination could be of benefit in Malaysia [18]. The second paper assessed the potential impact of 7-valent pneumococcal conjugate vaccine (PCV7) on the clinical and economic burden of pneumococcal disease in children in 2006–2007, concluding that PCV7 would be cost effective using the WHO thresholds for cost effectiveness [19]. This latter analysis was based on annual incidence per 1,000 population (from estimates of adults ≥ 20 years): 0.108 for pneumococcal meningitis; 7.784 for pneumococcal pneumonia; and 3.278 for pneumococcal bacteremia. The PCV13 vaccine serotype distribution in Malaysia before the introduction of PCV into the National Immunization Program has been documented from publications that detailed serotyping of invasive pneumococcal disease isolates over the period from 1995 to 2019. The PCV13 serotype coverage ranged from 76.0 to 87.6% for children (less than 5 years of age), 77.3% for adults (greater than 50 years of age), and it was from 51.4 to 77.7% for all ages [20–26]. This current analysis of data extracted from MoH facilities adds to the published evidence for microbiologically confirmed pneumococcal disease in the Malaysian population.

Nonetheless, this study has some limitations. Most importantly, it should be noted that though the diagnostic ICD-10 codes for pneumococcal diseases were the most reliable measures available, their use would inevitably have resulted in significant underreporting of the pneumococcal pneumonia disease burden. For instance, identification of the pneumonia pathogen is infrequent because most pneumonia is not bacteremic, even in cases of radiologically confirmed pneumonia. Non-bacteremic pneumonia seems to account for a sizable proportion of the disease burden, particularly in adults as compared to young children [27]. For the more serious conditions such as meningitis, in contrast, the magnitude to which these factors affected estimations of the burden of pneumococcal disease in this study is likely less because laboratory investigations would almost certainly have been more extensive. The database did not specify whether the diagnosis associated with hospitalization was the primary or secondary reason for the hospitalization. Other possible limitations associated with any analysis of retrospective database information could also apply to this study, including miscoding of disease episodes, changes in case definitions and testing regimens over time, and incomplete data. Although patients in Malaysia have a unique identifier, individuals could have been counted more than once because repeat admissions for the same episode are not differentiated within the MyHDW database.

Since the period covered by this study, Malaysia included PCV in its NIP in December 2020 for children below 2 years of age, born since 1 January 2020 [10]. This was in accordance with World Health Organisation (WHO) recommendations that PCVs be included in NIPs worldwide, especially in countries with a large childhood mortality burden [28]. Data on the impact of the NIP on pneumococcal disease in Malaysia are awaited. Adult immunisation with both 13-valent pneumococcal conjugate vaccine (PCV13) and 23-valent pneumococcal polysaccharide vaccine (PPSV23) in older adults and high-risk groups is recommended by the Malaysian Society of Infectious Diseases and Chemotherapy [29].

## Conclusions

The major advantage of database studies, especially for developing countries, is that they are far less resource intensive. Data quality is not as robust as that collected through prospective primary data collection; therefore, a prospective study applying enhanced testing protocols would allow estimation of the proportion of cases not captured in a retrospective database study. Nonetheless, the data presented here demonstrate that, in keeping with the Southeast Asia region, Malaysia experiences a significant burden from invasive pneumococcal disease (meningitis, pneumonia, or non-meningitis non-pneumonia). Measures to prevent infection, including vaccination, are likely be of benefit.

### Supplementary Information

**Additional file 1: Table S1. **Crude incidence rates of pneumococcal meningitis^1^, pneumococcal pneumonia^2^, and non-meningitis non-pneumonia pneumococcal disease^3^ per 100,000 (95% CI) by geographic region, Malaysia 2013-2015. **Table S2.** Crude mortality rates of pneumococcal meningitis^1^, pneumococcal pneumonia^2^, and non-meningitis non-pneumonia pneumococcal disease^3^ per 100,000 (95% CI) by geographic region and age group, Malaysia 2013-2015.

## Data Availability

All data generated or analysed during this study are included in this published article and its supplementary information files.

## Abbreviations

IPD: invasive pneumococcal disease
AOM: acute otitis media
MoH: Ministry of Health
MyHDW: Malaysian Health Data Warehouse
ICD-10: International Classification of Diseases, 10th Revision
NIP: national immunization programme
PCV: pneumococcal conjugate vaccine
PCV7: 7-valent pneumococcal conjugate vaccine
PCV10: 10-valent pneumococcal conjugate vaccine
PCV13: 13-valent pneumococcal conjugate vaccine
PPSV23: 23-valent pneumococcal polysaccharide vaccine
WHO: World Health Organization
