# Supplementary material for: Retrospective database analysis for clinical diagnoses commonly associated with pneumococcal diseases in the Malaysian healthcare system over a 3-year period (2013–2015)
Source: BMC Infect Dis. 2024 Jan 12;24:79. doi: 10.1186/s12879-023-08611-3 (PMC10790256; doi:10.1186/s12879-023-08611-3)
Supplement: Supplementary file 1 — Additional file 1: Table S1. Crude incidence rates of pneumococcal meningitis1, pneumococcal pneumonia2, and non-meningitis non-pneumonia pneumococcal disease3 per 100,000 (95% CI) by geographic region, Malaysia 2013-2015. Table S2. Crude mortality rates of pneumococcal meningitis1, pneumococcal pneumonia2, and non-meningitis non-pneumonia pneumococcal disease3 per 100,000 (95% CI) by geographic region and age group, Malaysia 2013-2015. [file 12879_2023_8611_MOESM1_ESM.docx]

| **Table S1. Crude incidence rates of pneumococcal meningitis^1^, pneumococcal pneumonia^2^, and non-meningitis non-pneumonia pneumococcal disease^3^ per 100,000 (95% CI) by geographic region, Malaysia 2013-2015** | | | | | | | |
| --- | --- | --- | --- | --- | --- | --- | --- |
| **Age (years)** | | | | | | | |
| **Region** | **<2 years** | **2 to <5 years** | **5 to <18 years** | **18 to <65 years** | **65 to <85 years** | **85 years+** | **All Ages** |
| **Pneumococcal meningitis** | | | | | | | |
| Northern^a^ | 0.35 (0.04, 1.25) | 0.24 (0.03, 0.86) | 0.05 (0.01, 0.18) | 0.01 (0, 0.05) | 0 (0, 0.28) | 0 (0, 3.79) | 0.04 (0.02, 0.08) |
| Central^b^ | 0.98 (0.42, 1.92) | 0 (0, 0.32) | 0.02 (0, 0.12) | 0.01 (0, 0.04) | 0 (0, 0.37) | 0 (0, 5.01) | 0.04 (0.02, 0.08) |
| Southern^c^ | 1.11 (0.41, 2.42) | 0.38 (0.08, 1.11) | 0.05 (0.01, 0.2) | 0.01 (0, 0.05) | 0 (0, 0.38) | 0 (0, 4.53) | 0.07 (0.04, 0.13) |
| East Coast^d^ | 1.25 (0.5, 2.58) | 0 (0, 0.46) | 0 (0, 0.11) | 0 (0, 0.05) | 0 (0, 0.53) | 0 (0, 6.22) | 0.05 (0.02, 0.11) |
| East^e^ | 1.48 (0.68, 2.8) | 0.53 (0.17, 1.23) | 0.07 (0.01, 0.19) | 0.05 (0.02, 0.11) | 0 (0, 0.48) | 0 (0, 5.33) | 0.12 (0.08, 0.18) |
| All regions | 1.03 (0.71, 1.46) | 0.22 (0.11, 0.41) | 0.04 (0.02, 0.08) | 0.02 (0.01, 0.03) | 0 (0, 0.08) | 0 (0, 0.97) | 0.06 (0.05, 0.08) |
| **Pneumococcal pneumonia** | | | | | | | |
| Northern^a^ | 2.6 (1.46, 4.3) | 0.36 (0.07, 1.05) | 0 (0, 0.09) | 0.03 (0.01, 0.09) | 0.22 (0.05, 0.65) | 1.03 (0.03, 5.72) | 0.14 (0.09, 0.2) |
| Central^b^ | 11.11 (8.95, 13.64) | 2.86 (1.97, 4.01) | 0.18 (0.08, 0.35) | 0.32 (0.24, 0.42) | 3.54 (2.47, 4.93) | 8.15 (2.99, 17.74) | 0.95 (0.83, 1.09) |
| Southern^c^ | 4.44 (2.85, 6.61) | 1.26 (0.61, 2.32) | 0.14 (0.04, 0.32) | 0.07 (0.03, 0.14) | 0.21 (0.03, 0.75) | 1.23 (0.03, 6.84) | 0.3 (0.22, 0.4) |
| East Coast^d^ | 2.51 (1.37, 4.21) | 1.01 (0.44, 1.99) | 0.11 (0.03, 0.29) | 0.22 (0.13, 0.35) | 0.71 (0.23, 1.66) | 0 (0, 6.22) | 0.36 (0.27, 0.48) |
| East^e^ | 4.76 (3.19, 6.83) | 0.84 (0.36, 1.66) | 0.13 (0.05, 0.29) | 0.1 (0.05, 0.17) | 1.29 (0.62, 2.37) | 1.45 (0.04, 8.05) | 0.35 (0.27, 0.44) |
| All Regions | 5.58 (4.78, 6.47) | 1.37 (1.05, 1.76) | 0 (0, 0.02) | 0.16 (0.13, 0.19) | 1.15 (0.87, 1.50) | 2.36 (1.08, 4.48) | 0.45 (0.41, 0.50) |
| **Non-meningitis non-pneumonia pneumococcal disease** | | | | | | | |
| Northern^a^ | 0.52 (0.11, 1.52) | 0.12 (0, 0.67) | 0.05 (0.01, 0.18) | 0.03 (0.01, 0.09) | 0.3 (0.08, 0.76) | 0 (0, 3.79) | 0.08 (0.04, 0.13) |
| Central^b^ | 1.95 (1.12, 3.17) | 0.52 (0.19, 1.13) | 0.04 (0.01, 0.16) | 0.18 (0.12, 0.26) | 3.14 (2.13, 4.45) | 9.51 (3.82, 19.6) | 0.39 (0.31, 0.48) |
| Southern^c^ | 0.56 (0.11, 1.62) | 0.25 (0.03, 0.91) | 0.05 (0.01, 0.2) | 0.09 (0.04, 0.17) | 0.73 (0.29, 1.5) | 1.23 (0.03, 6.84) | 0.15 (0.09, 0.22) |
| East Coast^d^ | 0.72 (0.2, 1.83) | 0.13 (0, 0.7) | 0.03 (0, 0.16) | 0.05 (0.01, 0.13) | 0.29 (0.03, 1.03) | 0 (0, 6.22) | 0.09 (0.05, 0.16) |
| East^e^ | 5.09 (3.46, 7.22) | 1.16 (0.58, 2.07) | 0.13 (0.05, 0.29) | 0.06 (0.02, 0.12) | 1.16 (0.53, 2.2) | 0 (0, 5.33) | 0.34 (0.26, 0.43) |
| All Regions | 1.84 (1.39, 2.38) | 0.46 (0.28, 0.71) | 0.07 (0.04, 0.12) | 0.1 (0.07, 0.13) | 1.15 (0.87, 1.50) | 0 (0, 0.97) | 0.23 (0.20, 0.27) |
| ^1^ICD-10 code: G00.1 (Pneumococcal meningitis) | | | | | | | |
| ^2^ICD-10 code: J13 (Pneumonia due to Streptococcus pneumoniae) | | | | | | | |
| ^3^ICD-10 code: B95.3 (Streptococcus pneumoniae as cause of diseases classified elsewhere), A40.3 (Sepsis due to Streptococcus pneumoniae) | | | | | | | |
| ^a^Kedah, Pulau Pinang, Perak | | | | | | | |
| ^b^Selangor, W.P. Kuala Lumpur, W.P. Putrajaya | | | | | | | |
| ^c^Negeri Sembilan, Melaka, Johor | | | | | | | |
| ^d^Kelantan, Terengganu, Pahang | | | | | | | |
| ^e^Sabah, Sarawak | | | | | | | |
| 95% CIs for proportions were calculated using exact method (Clopper-Pearson) | | | | | | | |

| **Table S2. Crude mortality rates of pneumococcal meningitis^1^, pneumococcal pneumonia^2^, and non-meningitis non-pneumonia pneumococcal disease^3^ per 100,000 (95% CI) by geographic region and age group, Malaysia 2013-2015** | | | | | | | |
| --- | --- | --- | --- | --- | --- | --- | --- |
| **Age (years)** | | | | | | | |
| **Region** | **<2 years** | **2 to <5 years** | **5 to <18 years** | **18 to <65 years** | **65 to <85 years** | **85 years+** | **All Ages** |
| **Pneumococcal meningitis** | | | | | | | |
| Northern^a^ | 0.17 (0, 0.97) | 0 (0, 0.44) | 0 (0, 0.09) | 0 (0, 0.03) | 0 (0, 0.28) | 0 (0, 3.79) | 0.01 (0, 0.3) |
| Central^b^ | 0.12 (0, 0.68) | 0 (0, 0.32) | 0 (0, 0.08) | 0 (0, 0.02) | 0 (0, 0.37) | 0 (0, 5.01) | 0 (0, 0.02) |
| Southern^c^ | 0 (0, 0.68) | 0 (0, 0.47) | 0.03 (0, 0.15) | 0.01 (0, 0.05) | 0 (0, 0.38) | 0 (0, 4.53) | 0.01 (0, 0.04) |
| East Coast^d^ | 0 (0, 0.66) | 0 (0, 0.46) | 0 (0, 0.11) | 0 (0, 0.05) | 0 (0, 0.53) | 0 (0, 6.22) | 0 (0, 0.03) |
| East^e^ | 0 (0, 0.61) | 0 (0, 0.39) | 0.02 (0, 0.12) | 0 (0, 0.03) | 0 (0, 0.48) | 0 (0, 5.33) | 0.01 (0, 0.03) |
| All Regions | 0.06 (0.01, 0.23) | 0 (0, 0.08) | 0.01 (0, 0.04) | 0 (0, 0.01) | 0 (0, 0.08) | 0 (0, 0.97) | 0.01 (0, 0.01) |
| **Pneumococcal pneumonia** | | | | | | | |
| Northern^a^ | 0 (0, 0.64) | 0 (0, 0.44) | 0 (0, 0.09) | 0 (0, 0.03) | 0.07 (0, 0.42) | 0 (0, 3.79) | 0.01 (0, 0.03) |
| Central^b^ | 0 (0, 0.45) | 0 (0, 0.32) | 0 (0, 0.08) | 0.04 (0.01, 0.08) | 0.71 (0.28, 1.46) | 4.08 (0.84, 11.91) | 0.07 (0.04, 0.11) |
| Southern^c^ | 0.19 (0, 1.03) | 0 (0, 0.47) | 0 (0, 0.1) | 0.01 (0, 0.05) | 0.1 (0, 0.58) | 0 (0, 4.53) | 0.02 (0, 0.05) |
| East Coast^d^ | 0 (0, 0.66) | 0 (0, 0.46) | 0 (0, 0.11) | 0.01 (0, 0.07) | 0 (0, 0.53) | 0 (0, 6.22) | 0.01 (0, 0.04) |
| East^e^ | 0.16 (0, 0.91) | 0 (0, 0.39) | 0 (0, 0.08) | 0.02 (0, 0.06) | 0 (0, 0.48) | 0 (0, 5.33) | 0.02 (0, 0.05) |
| All Regions | 0.06 (0.01, 0.23) | 0 (0, 0.08) | 0 (0, 0.02) | 0.02 (0.01, 0.03) | 0.19 (0.09, 0.36) | 0.79 (0.16, 2.3) | 0.03 (0.02, 0.04) |
| **Non-meningitis non-pneumonia pneumococcal disease** | | | | | | | |
| Northern^a^ | 0.17 (0, 0.97) | 0 (0, 0.44) | 0 (0, 0.09) | 0.01 (0, 0.05) | 0.07 (0, 0.42) | 0 (0, 3.79) | 0.02 (0, 0.05) |
| Central^b^ | 0.12 (0, 0.68) | 0 (0, 0.32) | 0 (0, 0.08) | 0.05 (0.02, 0.1) | 1.52 (0.85, 2.5) | 9.51 (3.82, 19.6) | 0.13 (0.09, 0.19) |
| Southern^c^ | 0 (0, 0.68) | 0.13 (0, 0.7) | 0 (0, 0.1) | 0.04 (0.01, 0.1) | 0.42 (0.11, 1.07) | 0 (0, 4.53) | 0.05 (0.03, 0.1) |
| East Coast^d^ | 0.36 (0.04, 1.29) | 0 (0, 0.46) | 0 (0, 0.11) | 0.01 (0, 0.07) | 0.14 (0, 0.79) | 0 (0, 6.22) | 0.03 (0.01, 0.08) |
| East^e^ | 0 (0, 0.61) | 0.11 (0, 0.59) | 0 (0, 0.08) | 0.02 (0.01, 0.07) | 0.9 (0.36, 1.86) | 0 (0, 5.33) | 0.06 (0.03, 0.1) |
| All Regions | 0.13 (0.04, 0.33) | 0.04 (0.01, 0.16) | 0 (0, 0.03) | 0.03 (0.02, 0.05) | 0.63 (0.42, 0.90) | 0 (0, 0.97) | 0.07 (0.05, 0.09) |
| ^1^ICD-10 code: G00.1 (Pneumococcal meningitis) | | | | | | | |
| ^2^ICD-10 code: J13 (Pneumonia due to Streptococcus pneumoniae) | | | | | | | |
| ^3^ICD-10 code: B95.3 (Streptococcus pneumoniae as cause of diseases classified elsewhere), A40.3 (Sepsis due to Streptococcus pneumoniae) | | | | | | | |
| ^a^Kedah, Pulau Pinang, Perak | | | | | | | |
| ^b^Selangor, W.P. Kuala Lumpur. W.P. Putrajaya | | | | | | | |
| ^c^Negeri Sembilan, Melaka, Johor | | | | | | | |
| ^d^Kelantan, Terengganu, Pahang | | | | | | | |
| ^e^Sabah, Sarawak | | | | | | | |
| 95% CIs for proportions were calculated using exact method (Clopper-Pearson) | | | | | | | |
